# Integrated Analysis of the RASH Study with the Use of the “Burden of Therapy” (BOTh^®TM^) Methodology—A Novel Tool for Assessing Adverse Events in Metastatic Pancreatic Cancer

**DOI:** 10.3390/curroncol30060436

**Published:** 2023-06-17

**Authors:** Klara Dorman, Stefan Boeck, Robert J. Snijder, Jens T. Siveke, Michael Schenk, Julia Mayerle, Karel Caca, Jens Freiberg-Richter, Ludwig Fischer von Weikersthal, Frank Kullmann, Anke Reinacher-Schick, Martin Fuchs, Stephan Kanzler, Volker Kunzmann, Thomas J. Ettrich, Danmei Zhang, Swantje Held, Ayad Abdul-Ahad, Michael von Bergwelt-Baildon, Volker Heinemann, Michael Haas

**Affiliations:** 1 Department of Medicine III and Comprehensive Cancer Center, University Hospital, Ludwig Maximilian University of Munich, Marchioninistr. 15, 81377 Munich, Germany; 2 German Cancer Consortium (DKTK), Partner Site Munich, 80336 Munich, Germany; 3 BOTh Analytics GmbH, 80687 Munich, Germany; 4 Bridge Institute of Experimental Tumor Therapy, West German Cancer Center, University Hospital Essen, 45147 Essen, Germany; 5 Division of Solid Tumor Translational Oncology, German Cancer Consortium (DKTK, Partner site Essen) and German Cancer Research Center, DKFZ, 69120 Heidelberg, Germany; 6 Department of Haematology and Oncology, Hospital Barmherzige Brüder, 93049 Regensburg, Germany; 7 Department of Internal Medicine II, University Hospital, LMU Munich, 81377 Munich, Germany; 8 Department of Internal Medicine I, Klinikum Ludwigsburg, 71640 Ludwigsburg, Germany; 9 Practice for Haematology and Oncology, 01307 Dresden, Germany; 10 Department of Oncology, Gesundheitszentrum St. Marien, 92224 Amberg, Germany; 11 Department of Medicine I, Klinikum Weiden, 92637 Weiden, Germany; 12 Department of Haematology and Oncology, St. Josef-Hospital, Ruhr University, 44791 Bochum, Germany; anke.reinacher@rub.de; 13 Department of Gastroenterology, Hepatology and Gastrointestinal Oncology, Klinikum Bogenhausen, 81925 Munich, Germany; 14 Department of Internal Medicine II, Leopoldina Krankenhaus Schweinfurt, 97422 Schweinfurt, Germany; 15 Department of Medical Oncology, University Hospital of Wuerzburg, 97080 Wuerzburg, Germany; 16 Department of Internal Medicine I, University of Ulm, 89081 Ulm, Germany; 17 ClinAssess GmbH, Department of Biometry, 51379 Leverkusen, Germany

**Keywords:** burden of therapy, erlotinib, FOLFIRINOX, gemcitabine, pancreatic cancer, quality of life, rash

## Abstract

This analysis of the RASH trial (NCT01729481) aimed at gaining a better understanding of the “Burden of Therapy” (BOTh^®TM^) in pancreatic ductal adenocarcinoma (PDAC). In the RASH study, 150 patients with newly diagnosed metastatic PDAC were treated with gemcitabine plus erlotinib (gem/erlotinib) for four weeks. Patients who developed a skin rash during this four-week run-in phase continued with the gem/erlotinib treatment, while rash-negative patients were switched to FOLFIRINOX. The study demonstrated a 1-year survival rate of rash-positive patients who received gem/erlotinib as first-line treatment that was comparable to previous reports of patients receiving FOLFIRINOX. To understand whether these comparable survival rates may be accompanied by better tolerability of the gem/erlotinib treatment compared to FOLFIRINOX, the BOTh^®TM^ methodology was used to continuously quantify and depict the burden of therapy generated by treatment emergent events (TEAEs). Sensory neuropathy was significantly more common in the FOLFIRINOX arm, and prevalence as well as severity increased over time. In both arms, the BOTh^®TM^ associated with diarrhea decreased over the course of treatment. The BOTh^®TM^ caused by neutropenia was comparable in both arms but decreased in the FOLFIRINOX arm over time, possibly due to chemotherapy dose reductions. Overall, gem/erlotinib was associated with a slightly higher overall BOTh^®TM^, but the difference was not statistically significant (*p* = 0.6735). In summary, the BOTh^®TM^ analysis facilitates the evaluation of TEAEs. In patients fit for intense chemotherapeutic regimens, FOLFIRINOX is associated with a lower BOTh^®TM^ than gem/erlotinib.

## 1. Introduction

**Figure 1:**
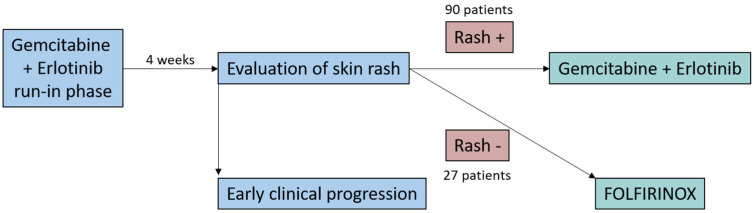
Study design of the RASH trial.

Pancreatic ductal adenocarcinoma (PDAC) remains one of the most lethal malignancies [1] and is projected to become the second leading cause of cancer-related death by 2030 [2]. Especially when treating metastatic disease, it is important to find the right balance between quantity and quality of life [3]. In patients fit enough for intense chemotherapeutic regimens, FOLFIRINOX (5-fluorouracil, folic acid, irinotecan, oxaliplatin) and gemcitabine/nab-paclitaxel are standard [4,5]. Patients not eligible for these regimens should be offered treatment with gemcitabine, which may be combined with the epidermal growth factor receptor (EGFR) inhibitor erlotinib [6,7]. The development of a skin rash under erlotinib has been shown to be associated with better survival [8,9]. The RASH study was a prospective, multicenter phase II trial conducted by the German “Arbeitsgemeinschaft Internistische Onkologie” (AIO) and aimed to evaluate whether patients fit for FOLFIRINOX who developed a skin rash during gem/erlotinib treatment might equally profit from the—at least according to the then available data—less toxic gem/erlotinib regimen instead of FOLFIRINOX (Figure 1). In a four-week run-in phase, 150 patients with newly diagnosed metastatic PDAC were treated with gem/erlotinib. Patients who developed a skin rash during these four weeks continued with the gem/erlotinib treatment, while 27 rash-negative patients were subsequently switched to FOLFIRINOX [10]. The study demonstrated a 1-year survival rate of rash-positive patients who received gem/erlotinib as first-line treatment that was comparable to previous reports of patients receiving FOLFIRINOX. FOLFIRINOX is typically regarded as an intense chemotherapeutic regimen applied to patients with good performance status [6]. With gemcitabine monotherapy being recommended as palliative chemotherapy in patients with metastatic pancreatic cancer and a reduced performance status, one might expect the combination gem/erlotinib to harbor less toxicity than FOLFIRINOX. A novel tool to investigate this hypothesis is the burden of therapy (BOTh^®TM^) method, which analyzes and visualizes the intensity and temporal evolution of treatment-emerging adverse events (TEAEs) during treatment. While abundant information on TEAEs is collected in clinical trials, this effort mostly results in merely one table depicting their frequency for each treatment arm without consideration of the duration of the TEAEs. To evaluate whether gem/erlotinib is in fact associated with a lower toxicity compared to FOLFIRINOX, the intensity and temporal evolution of TEAEs during treatment in the RASH trial were analyzed and compared using the BOTh^®TM^ method.

## 2. Materials and Methods

The trial’s design, along with the efficacy and safety data from the RASH study, have been extensively detailed in a previous publication [10]. To delve deeper into the TEAEs experienced by all patients on a weekly basis, we undertook a retrospective analysis of data collected from 150 patients included in the RASH study. This analysis was conducted using the innovative BOTh^®TM^ methodology, comprehensively described by Abdulahad and colleagues [11]. The BOTh^®TM^ methodology determines a weekly “burden estimate” based on the number and severity of reported TEAEs. This estimate is graphically represented via a chart generated by the SAS 9.4 software. To illustrate, consider a scenario where a patient experiences grade 2 diarrhea and a grade 1 skin rash within the same week. This patient’s BOTh for that week would be calculated as the sum of the products of each adverse event grade and its presence indicator, in this case: (2 × 1) + (1 × 1) = 3. When a patient reported the same TEAE with varying severity within the same week, the highest severity was used for the analysis. When a TEAE was reported as ongoing at the end of the study, the patient’s last study day was treated as the end date of the TEAE. Each vertical bar on the BOTh^®TM^ graph reflects the total burden of all study subjects in a specific week divided by the subjects at risk during that time period. The total burden of therapy for each treatment arm can be quantified by calculating the area under the curve (AUC). The AUC symbolizes the total accumulated BOTh over time, providing a basis for statistical comparison between different treatment arms [11].

## 3. Results

**Figure 2:**
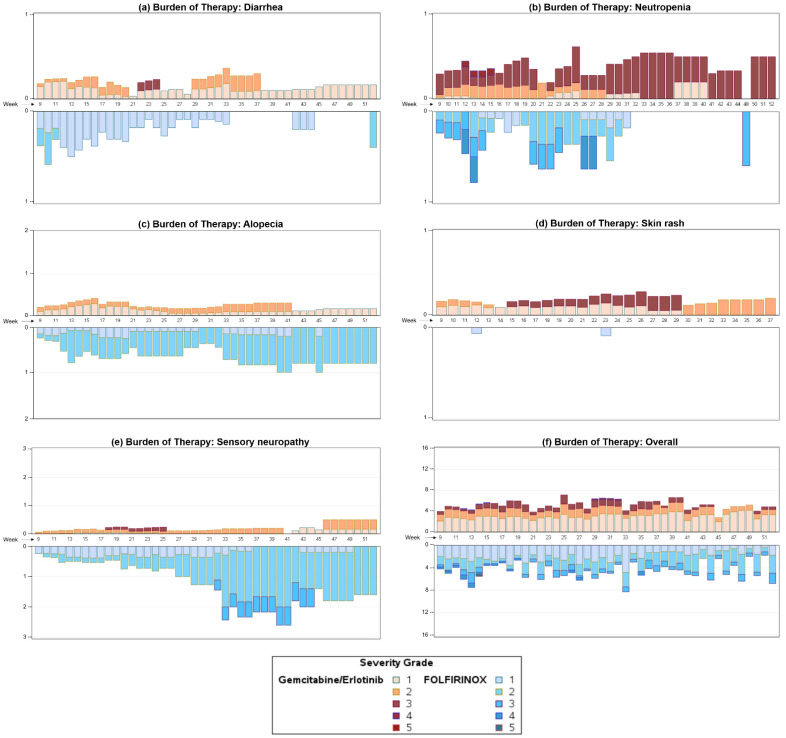
BOTh^®TM^ graph of diarrhea (**a**), neutropenia (**b**), alopecia (**c**), skin rash (**d**), sensory neuropathy (**e**), and overall burden of therapy (**f**) in the RASH trial. The graph is a mirrored display bar chart with the *y*-axis showing the study week and the *x*-axis displaying the number of TEAEs, allowing comparison of the burden of therapy per week of each treatment over the course of the clinical trial. Varied weighting has been applied to TEAEs depending on the reported severity, and coloring corresponds to TEAE severity.

We analyzed the overall burden of therapy and the burden caused by the common TEAEs skin rash, sensory neuropathy, alopecia, diarrhea, and neutropenia in the phase II RASH trial (Figure 2). As expected, the occurrence of skin rash was significantly more common in the gem/erlotinib arm (*p* < 0.0001), whereas the burden of therapy caused by sensory neuropathy was significantly higher in the FOLFIRINOX arm (*p* = 0.0048). The BOTh^®TM^ graph clearly depicts the increase in sensory neuropathy severity over time, not only in prevalence but also in severity. While alopecia was a frequent burden in both treatment arms, it seemed more common in the FOLFIRINOX arm; however, this difference was not statistically significant. Interestingly, diarrhea was a common TEAE in both arms, but the burden decreased in the FOLFIRINOX arm over time while it remained fairly stable in the gem/erlotinib arm. Neutropenia, an important TEAE often related to treatment interruptions or infections, was analyzed as well. While there was no clear difference in the burden of therapy for this aspect (*p* = 0.8), it became apparent that the burden of therapy associated with neutropenia decreased, particularly pronounced in the FOLFIRINOX arm over time, while it remained a relevant TEAE in the gem/erlotinib arm. In the overall comparison of the burden of therapy in the two treatment arms, no statistically significant difference was observed (*p* = 0.6735). However, there seemed to be a tendency towards a higher burden of therapy in the gem/erlotinib arm. The burden of therapy remained relatively stable over time in both arms, with only the FOLFIRINOX arm showing a higher week-to-week variation in the later phase of the trial. The occurrence of severe TEAEs seemed to be distributed equally not only among the two treatment arms but also over the course of the study.

## 4. Discussion

Despite extensive efforts towards identifying innovative treatment options, the prognosis for patients diagnosed with PDAC remains poor [12]. Especially in metastatic disease, this increases the importance of quality of life for patients. Aside from tumor-associated symptoms such as pain, cachexia, depression, and ascites [13], TEAEs may also influence quality of life. As symptoms associated with the disease itself are to be addressed by a multidisciplinary team [13], the same should apply to TEAEs. A major challenge, especially in palliative settings, remains finding the optimal balance between lowering the burden of disease through therapy and not negatively impacting quality of life due to TEAEs. The BOTh^®TM^ analysis provides a useful tool in understanding the temporal evolution of the burden of therapy. This way, the benefit for the patient can be determined more accurately and in relation to time. BOTh^®TM^ has been proposed as the new standard for analyzing safety in clinical trials [11]. Especially in the future, it becomes increasingly important to identify a potential gap between therapeutic benefit and therapeutic burden in order to spare patients unnecessarily intense treatments.

The RASH trial aimed at evaluating whether a selected group of patients (qualifying for therapy with FOLFIRINOX and developing a skin rash due to erlotinib) might equally profit from the—at least according to the then available data—less toxic gem/erlotinib regimen compared to FOLFIRINOX.

As expected, the FOLFIRINOX arm was associated with a significantly higher rate of polyneuropathy, most probably attributed to oxaliplatin. The intensity clearly correlated with the length of treatment, while this side effect hardly affected patients on gem/erlotinib in a significant manner. A slightly higher rate of alopecia becomes apparent in FOLFIRINOX-treated patients, while skin rash is clearly associated with erlotinib treatment.

We would have expected a higher rate of diarrhea in the FOLFIRINOX-treated patients (due to irinotecan), but, surprisingly, the burden of therapy seems to be equal during both regimens, being higher in the first phase of FOLFIRINOX treatment but then resolving, probably due to dose reductions. By contrast, in the gem/erlotinib group, diarrhea seemed to peak after several weeks of treatment.

A similar observation can be made for neutropenia, which we had expected to be more severe in the FOLFIRINOX arm but which overall seemed to be equally manageable compared to treatment with gem/erlotinib.

The overall burden of therapy was even slightly higher in the gem/erlotinib treated patients compared to FOLFIRINOX, though not statistically significant. This result represents a clear contrast to the assumptions on tolerability of both regimens at the time when the trial was designed, but is in line with the quality of life analysis of the PRODIGE 4 trial, which investigated FOLFIRINOX versus gemcitabine monotherapy, where FOLFIRINOX significantly improved quality of life overall [14]. Although rash-negative patients had a lower quality of life at baseline, a lower proportion of patients in this group reached the end-point “time to definitive deterioration ≥20 points for the global health status—QL2” [10].

The randomized phase II study ACCEPT—a clinical trial also from our AIO study group—evaluated gemcitabine plus afatinib versus gemcitabine alone in metastatic PDAC and has already been retrospectively analyzed with the BOTh^®TM^ method [15]. It could be shown that the overall burden of therapy was higher in the gemcitabine plus afatinib arm; however, the visualization of TEAEs in the BOTh^®TM^ graph revealed that day-to-day variations were more pronounced in the monotherapy arm. Information like this would not be visible in a standard table depicting the proportion of patients experiencing a certain AE in a clinical trial. The BOTh^®TM^ analysis therefore aims to make the best use of the vast amounts of data collected in clinical trials.

In an effort to define recommended and mandatory parameters that should be included in clinical trials for PDAC, Ter Veer and colleagues have labeled 23 baseline characteristics as mandatory and 12 as recommended [16]. The consensus statement had the goal of standardizing clinical trials in PDAC and defining the minimal information that should be evaluated in all studies. Quality of life analyses were recommended for all clinical trials investigating treatment options for PDAC. Maharaj and colleagues have identified the FACT HEP questionnaire as the best patient-reported outcome measure for unresectable PDAC in a systematic review [17]. The FACT HEP questionnaire is validated disease-specifically for PDAC, multidimensional, and shows good responsiveness. In the future, further improvement of comparability and objectivity in clinical trial reporting may be achieved through the implementation of the BOTh^®TM^ analysis in clinical trials investigating PDAC. Combining patient reported outcome measures with the BOTh^®TM^ analysis in clinical trials might enhance the understanding of the influence of TEAEs on the patient’s wellbeing.

A weakness of the current analysis is the possibly reduced validity of the results at later timepoints in the trial. Since fewer patients are receiving treatment within the trial over time, the BOTh^®TM^ graph reflects the burden of therapy for a continuously decreasing number of patients. This way, the TEAEs of individual patients have a higher impact and complicate the interpretation. Additionally, some TEAEs can falsely appear to decrease over time, when in fact this might be due to dose reductions or due to patients with severe TEAEs ending the study treatment. Another difficulty is that some TEAEs, such as diarrhea, are not only caused by the treatment but are also influenced by the underlying disease itself. Furthermore, the TEAEs that are analyzed within the overall BOTh^®TM^ are not weighted differently depending on their clinical relevance, which can weaken the informative value of the overall analysis from a clinical standpoint. Future analyses of clinical trials with the BOTh^®TM^ method might implement a weighting system depending on the clinical relevance of the different TEAEs to depict the clinical impact more accurately.

Despite these remaining challenges, the BOTh^®TM^ method provides a useful tool to evaluate TEAEs in clinical studies. To make the most of the copious amounts of data collected in clinical trials, new methods such as the BOTh^®TM^ method can be combined with quality of life assessments and conventional efficacy reporting. The BOTh^®TM^ graph makes time-dependent differences in TEAEs apparent, helps to accurately convey clinically relevant findings, and may facilitate better treatment choices, especially in a palliative setting.

## Data Availability

Available from the corresponding author upon reasonable request.

## Presentation

Parts of this manuscript were presented as a poster at the DGHO congress 2022.

## References

[1] Siegel R.L., Miller K.D., Fuchs H.E., Jemal A. (2021). Cancer Statistics, 2021. CA Cancer J. Clin..

[2] Rahib L., Smith B.D., Aizenberg R., Rosenzweig A.B., Fleshman J.M., Matrisian L.M. (2014). Projecting cancer incidence and deaths to 2030: The unexpected burden of thyroid, liver, and pancreas cancers in the United States. Cancer Res..

[3] Warner-Cohen J., Saif M.W. (2020). Delicate Balance Between Quality and Quantity of Life: Palliative Chemotherapy for Metastatic Pancreatic Adenocarcinoma. JCO Oncol. Pr..

[4] Conroy T., Desseigne F., Ychou M., Bouché O., Guimbaud R., Bécouarn Y., Adenis A., Raoul J.L., Gourgou-Bourgade S., de la Fouchardière C. (2011). FOLFIRINOX versus gemcitabine for metastatic pancreatic cancer. N. Engl. J. Med..

[5] Von Hoff D.D., Ervin T., Arena F.P., Chiorean E.G., Infante J., Moore M., Seay T., Tjulandin S.A., Ma W.W., Saleh M.N. (2013). Increased survival in pancreatic cancer with nab-paclitaxel plus gemcitabine. N. Engl. J. Med..

[6] Sohal D.P.S., Kennedy E.B., Cinar P., Conroy T., Copur M.S., Crane C.H., Garrido-Laguna I., Lau M.W., Johnson T., Krishnamurthi S. (2020). Metastatic Pancreatic Cancer: ASCO Guideline Update. J. Clin. Oncol..

[7] Moore M.J., Goldstein D., Hamm J., Figer A., Hecht J.R., Gallinger S., Au H.J., Murawa P., Walde D., Wolff R.A. (2007). Erlotinib plus gemcitabine compared with gemcitabine alone in patients with advanced pancreatic cancer: A phase III trial of the National Cancer Institute of Canada Clinical Trials Group. J. Clin. Oncol..

[8] Heinemann V., Vehling-Kaiser U., Waldschmidt D., Kettner E., Marten A., Winkelmann C., Klein S., Kojouharoff G., Gauler T.C., von Weikersthal L.F. (2013). Gemcitabine plus erlotinib followed by capecitabine versus capecitabine plus erlotinib followed by gemcitabine in advanced pancreatic cancer: Final results of a randomised phase 3 trial of the ‘Arbeitsgemeinschaft Internistische Onkologie’ (AIO-PK0104). Gut.

[9] Van Cutsem E., Vervenne W.L., Bennouna J., Humblet Y., Gill S., Van Laethem J.L., Verslype C., Scheithauer W., Shang A., Cosaert J. (2009). Phase III trial of bevacizumab in combination with gemcitabine and erlotinib in patients with metastatic pancreatic cancer. J. Clin. Oncol..

[10] Haas M., Siveke J.T., Schenk M., Lerch M.M., Caca K., Freiberg-Richter J., Fischer von Weikersthal L., Kullmann F., Reinacher-Schick A., Fuchs M. (2018). Efficacy of gemcitabine plus erlotinib in rash-positive patients with metastatic pancreatic cancer selected according to eligibility for FOLFIRINOX: A prospective phase II study of the ‘Arbeitsgemeinschaft Internistische Onkologie’. Eur. J. Cancer.

[11] Abdulahad A.K., Snijder R.J., Panni M.K., Riaz F.K., Karas A.J. (2016). A novel standard to evaluate the impact of therapeutic agents on patient safety—The BURDEN OF THERAPY™©*. Contemp. Clin. Trials Commun..

[12] Dorman K., Heinemann V., Kobold S., von Bergwelt-Baildon M., Boeck S. (2022). Novel systemic treatment approaches for metastatic pancreatic cancer. Expert. Opin. Investig. Drugs.

[13] Lee K.G., Roy V., Laszlo M., Atkins K.M., Lin K.J., Tomassian S., Hendifar A.E. (2021). Symptom Management in Pancreatic Cancer. Curr. Treat. Options Oncol..

[14] Gourgou-Bourgade S., Bascoul-Mollevi C., Desseigne F., Ychou M., Bouche O., Guimbaud R., Becouarn Y., Adenis A., Raoul J.L., Boige V. (2013). Impact of FOLFIRINOX compared with gemcitabine on quality of life in patients with metastatic pancreatic cancer: Results from the PRODIGE 4/ACCORD 11 randomized trial. J. Clin. Oncol..

[15] Haas M., Waldschmidt D.T., Stahl M., Reinacher-Schick A., Freiberg-Richter J., Fischer von Weikersthal L., Kaiser F., Kanzler S., Frickhofen N., Seufferlein T. (2021). Afatinib plus gemcitabine versus gemcitabine alone as first-line treatment of metastatic pancreatic cancer: The randomised, open-label phase II ACCEPT study of the Arbeitsgemeinschaft Internistische Onkologie with an integrated analysis of the ‘burden of therapy’ method. Eur. J. Cancer.

[16] Ter Veer E., van Rijssen L.B., Besselink M.G., Mali R.M.A., Berlin J.D., Boeck S., Bonnetain F., Chau I., Conroy T., Van Cutsem E. (2018). Consensus statement on mandatory measurements in pancreatic cancer trials (COMM-PACT) for systemic treatment of unresectable disease. Lancet Oncol..

[17] Maharaj A.D., Samoborec S., Evans S.M., Zalcberg J., Neale R.E., Goldstein D., Merrett N., White K., Croagh D., Pilgrim C.H.C. (2020). Patient-reported outcome measures (PROMs) in pancreatic cancer: A systematic review. HPB.

